# Supplementary material for: The magnitude of non-adherence and contributing factors among adult outpatient with Diabetes Mellitus in Dilla University Referral Hospital, Gedio, Ethiopia
Source: PLoS One. 2021 Mar 4;16(3):e0247952. doi: 10.1371/journal.pone.0247952 (PMC7932062; doi:10.1371/journal.pone.0247952)

Fig-1 showing the magnitude of adherence and non-adherence among the participants among T2DM patients who attend the medical referral clinics for treatment and follow up at DURH, 2017

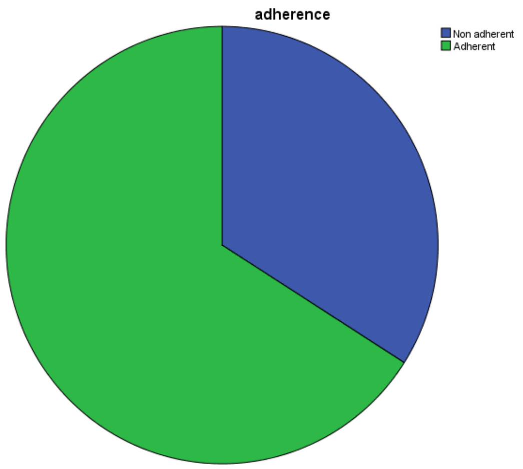

Supplement: S1 Fig — (PDF) [file pone.0247952.s001.pdf]
