# Supplementary material for: The magnitude of non-adherence and contributing factors among adult outpatient with Diabetes Mellitus in Dilla University Referral Hospital, Gedio, Ethiopia
Source: PLoS One. 2021 Mar 4;16(3):e0247952. doi: 10.1371/journal.pone.0247952 (PMC7932062; doi:10.1371/journal.pone.0247952)
Supplement: S1 File — (PDF) [file pone.0247952.s002.pdf]

Annex I: DILLA UNIVERSITY, COLLEGE OF HEALTH SCIENCE, SCHOOL MEDICINE

Questionnaires on Sociodemographic characteristics, adapted tool based on eight item Morisky Medication adherence questionnaire (1), contributing factors & Key Informant Interview Guide on Diabetes mellitus in Dilla University Referral Hospital.

Instruction: This questionnaire is designed to assess non adherence and contributing factors in Diabetes mellitus patients. The result of this study was utilized for planning health service in the study area; you are politely requested to respond the questionnaires. You response was secrete

PART ONE: SOCIODEMOGRAPHIC CHARACTRASTIC

Instruction: PUT '√' MARK AGAINST THE ANSWER THAT THE PATIENT THINK CORRECT FOR HIMSELF

1. Age in year**…………**

2. Sex: male…… female……..

3. Religion A. Orthodox ------------- C. Protestant------------

B Muslim -------------- D. Catholic----------- E. others-----------

4. Educational status

A. illiterate ------------ D. Grade 9-12 ------------

B. read and write ------------- E. Technique and vocation ------

C. Grade 1-8 -------------- F. College/ University ------------

5. Martial status

A. Single ------------ B. Married-------- C. Widowed-------- D. divorced------

6. Occupation

A. Governmental employee --------------- D. Merchant-------------

B. Private working-------------- E. House wife--------------

C. Student -------------- F. Others specify --------------

7. Monthly income in birr A. <150----- B. 150-500----C.501-1000--- D. >1000

8. Type of diabetes Type I--------- Type II---------

9. Social support from friends and others A. Yes----- B. NO ------

10. If yes for question specify------

11. Do you exercise every day? 1. Yes 2. No

12. If yes for the question how long do you exercise per day __________ in minutes?

13. If no for the question no. 12 specify the reason ________________________________

14. Do you follow the diet advised by your Doctor? 1. Yes 2. No

15. if no for the question no. 14 specify the reason ________________________________

PART-TWO Adapted version of eight item Morisky Medication adherence questionnaire (1)

1. Do you sometimes forget to take your drug

1. Yes 2. No

2. Thinking over the past 2 weeks, were there any days when you did not take your drugs

   1. Yes 2. No

3. Have you ever stopped taking your medicine without telling your doctor because you felt worse when you took it

   1. Yes 2. No

4. When you travel or leave home, do you sometimes forget to bring along your drugs

   1. Yes 2. No

5. Did you take all your latest dose of your drugs

   1. Yes 2. No

6. When you feel like your symptoms are under control, do you sometimes stop taking your drugs

   1. Yes 2. No

7. Taking medicine every day is difficult for some people, Do you ever feel pressurized about sticking to your treatment plan

   1. Yes 2. No

8. How often do you have difficulty remembering to take all your drugs,

   (A) Never/rarely, (B) Once in a while, (C) Sometimes, (D) Usually, (E) All the time

PART -3 **Factors contributing with non-adherence to treatment regimen**

1. How long ago were you told you had diabetes? ____________ Years

2. How long have you been on ant diabetic medication? ____________Months

3. Do you sometimes have problems remembering to take your diabetic medication at the assigned / Right time? Yes…… No …….

4. When you feel better, do you sometimes stop taking your diabetic medication?

Yes…… No…….

5. Sometimes when you feel worse when you take diabetic medication, do you stop taking it?

Yes…… No…….

6. What do u think the cause for diabetes mellitus?

7. People miss taking their medication for various reasons. What reasons sometimes make you miss? Taking your diabetic medication as prescribed? **(You can tick more than one response)**

	**Possible Reasons**	**No = 0**	**Yes =1**
A	When you are busy with other things?		
B	When you have too many pills to take?		
C	When you want to avoid side effects?		
D	When you are upset/ depressed or overwhelmed?		
E	When you ran out of pills?		
F	When you cannot afford the drugs?		
G	When you are away from home?		
H	When you take alcohol/other substance use?		

I	When you do not have money for transport to go to the [illegible]		
J	When you do not understand how to take diabetic medication?		
K	When you do not want others to notice that you are taking diabetic medication?		
L	When you feel that taking ant diabetic medication has no benefit		
M	When you feel that taking ant diabetic medication harms your health		
N	Others (specify)		

8. Did you get **all** the diabetic medication(s) that you are currently on from Dilla University Referral?

Hospital pharmacy?

Yes ……… No ……….

9. **IF NO, to Q.7** why? ____

10. How far do you live from the health facility where you collect your diabetic medication in kilometers? __________________ Kilometers

11. Do you find it difficult asking health care providers (Doctors, nurses, pharmacists) questions about

diabetes and its treatment? Yes ………. No…………

12. Have you ever received diabetic education? Yes………… No………………………

13. **IF YES**, to no. 11, how long ago did you receive the last diabetic education? __________Months

14. Do you know the benefits of taking your diabetic medicine as told to you by health care provider?

Yes ……… No...………

15. Are you on any other medication for other long term illnesses? (Illnesses lasting for more than 3 Months) No……….. Yes………

16. Have you ever been told by a health care provider that you have any of the following problems, eye, Heart, high blood pressure, kidney or feet problem? Yes ……… No ………

17. Do those around you and your family encourage you to take your diabetes medication?

Yes ……… No………

18. Do you sometimes take traditional medicine for managing diabetes?

Yes …. No ……

19. Have you experienced any side effects with your prescribed Drugs? Yes…….. No……………………

20. If yes, then describe?

21. Have you been asked to report if you develop any side effects?

Yes……… No……

22. How frequently do you get your blood glucose checked? Yes ……………No……………

23. Is it self-monitoring or at lab? Yes………….No………………

24. Is your sleep disturbed too much or u have broken sleep?

Yes …………… No……………..

Thank you for your time and for participating in this study

PART 4: Key Informant Interview Guide

Interview Guide Number __ __ ___

Respondent Title ____________________

Date of interview __ __/ __ __ / __ __

Q1. Please tell me about adherence to ant diabetic treatment among diabetic patients
Attending your clinic?

Q2. How does non-adherence to anti-diabetic treatment among diabetic out patients
Attending your clinic?

Q3. Approximately, what is the proportion of those who do not adhere to ant diabetic treatment?

Q4. What are the patients' social perceptions surrounding diabetes mellitus and its treatment?

Q5. What are the reasons for not adhering to ant diabetic treatment as reported to you by the
Patients?

Q6. In your view, how does health system within which you operate, contributes to non-adherence to
Ant diabetic treatment among diabetic patients attending your clinic?

Q7. What else would you like bring up in relation to non-adherence to ant diabetic treatment among?
Diabetic out patients attending your clinic?

Thank you for taking time to participate in this interview
