# Supplementary material for: The magnitude of non-adherence and contributing factors among adult outpatient with Diabetes Mellitus in Dilla University Referral Hospital, Gedio, Ethiopia
Source: PLoS One. 2021 Mar 4;16(3):e0247952. doi: 10.1371/journal.pone.0247952 (PMC7932062; doi:10.1371/journal.pone.0247952)
Supplement: Attachment — Submitted filename: Review comments.docx [file pone.0247952.s003.docx]

Reviewer comments for the manuscript titled “ *Assessing the Magnitude of Non-adherences to the treatment of DM and associated factors ; A hospital based Cross-sectional study*”

The authors have made interesting observations in this study. Nevertheless, the study is demerited for several reasons as detailed in my comments below.

1. The study is not powered by a statistically calculated sample size as it has applied random sampling method. The observations of the study cannot be extrapolated to the general population due to it’s cross-sectional design.
2. The study was conducted in 2017 and is communicated to a journal in 2020. This is a major concern as it limits the validity of the results for the recent times. The sample size is 191 subjects. To conduct this study in a hospital setting should not have been time consuming. I am concerned for reasons in delay in conducting and completing the study.
3. The salient findings of the study as mentioned in the abstract do not add any novel dimension for further research. I do not find any significance except for the fact that it is reported from a hospital in Ethiopia.
4. The manuscript is badly formatted. It appears that the authors did not proof check the manuscript and did not adhere to neat formatting norms on Word document. The manuscript appears un-appealing to the reviewers.
5. The authors have used erroneous English with too many grammatical errors and incomplete sentences. For eg *“* In the section on introduction the line *“In Africa, up to 80 % of diabetic patients are underdiagnosed and will complicate*” I am sure that this manuscript was not proof checked.
6. The reference for Morisky Medication adherence questionnaire has not been cited.
7. Though the tables 1 and 2 have been cited, they do not appear in the main PDF of the manuscript. The tables are badly formatted and do not appeal to the reviewers.
8. There are no foot notes below tables which indicate the lapse in correct presentation of results.
9. Figures 1 and 2 are shoddy and needless.
10. References are badly formatted and do not comply to the journal’s specifications.
11. References 1 is unsuitable for a citation.
12. In short, this is a shoddy presentation of cross-sectional study with no robust validation. It would not worthwhile to make technical comments for a manuscript which does not comply to the basic requirements of a scientific manuscript. The authors need to seriously review the standard of writing manuscripts before communicating them to an international peer reviewed journal of significant impact factor. In view of the comments above, I deem it unsuitable for publication in Plos One in the present form.
